# Comparison of the Dynesys Dynamic Stabilization System and Posterior Lumbar Interbody Fusion for Lumbar Degenerative Disease

**DOI:** 10.1371/journal.pone.0148071

**Published:** 2016-01-29

**Authors:** Yang Zhang, Jian-Lin Shan, Xiu-Mei Liu, Fang Li, Kai Guan, Tian-Sheng Sun

**Affiliations:** Department of Orthopedics, Beijing Army General Hospital, Beijing 100700, China; University of Michigan, UNITED STATES

## Abstract

**Background:**

There have been few studies comparing the clinical and radiographic outcomes between the Dynesys dynamic stabilization system and posterior lumbar interbody fusion (PLIF). The objective of this study is to compare the clinical and radiographic outcomes of Dynesys and PLIF for lumbar degenerative disease.

**Methods:**

Of 96 patients with lumbar degenerative disease included in this retrospectively analysis, 46 were treated with the Dynesys system and 50 underwent PLIF from July 2008 to March 2011. Clinical and radiographic outcomes were evaluated. We also evaluated the occurrence of radiographic and symptomatic adjacent segment degeneration (ASD).

**Results:**

The mean follow-up time in the Dynesys group was 53.6 ± 5.3 months, while that in the PLIF group was 55.2 ± 6.8 months. At the final follow-up, the Oswestry disability index and visual analogue scale score were significantly improved in both groups. The range of motion (ROM) of stabilized segments in Dynesys group decreased from 7.1 ± 2.2° to 4.9 ± 2.2° (*P* < 0.05), while that of in PLIF group decreased from 7.3 ± 2.3° to 0° (*P* < 0.05). The ROM of the upper segments increased significantly in both groups at the final follow-up, the ROM was higher in the PLIF group. There were significantly more radiographic ASDs in the PLIF group than in the Dynesys group. The incidence of complications was comparable between groups.

**Conclusions:**

Both Dynesys and PLIF can improve the clinical outcomes for lumbar degenerative disease. Compared to PLIF, Dynesys stabilization partially preserves the ROM of the stabilized segments, limits hypermobility in the upper adjacent segment, and may prevent the occurrence of ASD.

## Introduction

Spinal fusion is considered the gold standard for treatment of spinal degenerative disease, although there are several complications associated with this technique, such as nonunion, instrumentation failure, infection, and pain in the donor area if an iliac bone graft is used. Moreover, increased range of movement (ROM) at adjacent segments after spinal fusion may increase the risk for adjacent segment degeneration (ASD) [1]. To avoid some of these undesirable effects, dynamic stabilization systems have been developed [2].

The Dynesys^®^ Dynamic Stabilization System (Zimmer Inc., Warsaw, IN, USA) is one of the most frequently used posterior dynamic stabilization devices [3]. The system is designed to stabilize the operated segment, while preserving some mobility, thus avoiding hypermobility of the adjacent segment. Many clinical studies performed over the past decade have reported positive outcomes in patients with lumbar degenerative disease treated with the Dynesys system [4, 5]. However, relatively few studies have compared clinical and radiographic outcomes between the Dynesys system and posterior lumbar interbody fusion (PLIF) [6]. In addition, long-term reports on the use of the Dynesys system in Chinese populations are comparatively rare. Therefore, the aim of this retrospective study was to compare the clinical and radiographic outcomes of the Dynesys system to those of PLIF for the treatment of lumbar degenerative disc disease in a Chinese population.

## Materials and Methods

### Patient selection

This retrospective study included 46 consecutive patients who underwent Dynesys stabilization for lumbar degenerative disc disease from July 2008 to March 2011. The study protocol was approved by the Institutional Review Board of our hospital. The approval number was 2015088. Because our study was retrospective and the records of patients were anonymized and de-identified prior to analysis, so we did not have consent from the participants. Fifty patients who underwent PLIF with the same disease at the same stage were retrospectively matched as a control group. The inclusion criteria and indications were: lumbar spinal stenosis with or without scoliosis (<10°) with degenerative disc disease and lumbar disc herniation with massive herniation with degenerative disc disease. The exclusion criteria were prior lumbar surgery, degenerative scoliosis >10°, severe osteoporosis (bone mineral density test T-score < –2.5), and severe spinal deformity.

All patients experienced failure of conservative treatment for at least 3 months before they were considered for surgical intervention. Every patient underwent pre-operative X-ray and magnetic resonance imaging (MRI) examinations. All patients were followed-up for a minimum of 48 months.

### Surgical technique

Stabilization with the Dynesys system was performed as an open procedure with a midline skin incision. General sub-dermal dissection through the same midline skin incision allowed another two fascial incisions at each side. The Dynesys screws were then placed transpedicularly via the Wiltse paraspinal approach without destruction of the facet joints. Standard laminectomies were performed cautiously to preserve the facet joints. However, for cases of severe stenosis or far lateral stenosis, extensive decompression was performed, during which the medial border of the superior facet was partially removed to provide a clear view of the involved nerve root. The constructs, polycarbonate-urethane spacers, and tension cords were assembled according to technical suggestions provided by the manufacturer. Postoperatively, patients in the Dynesys group wore a soft lumbar brace for 3 months [7].

PLIF was performed with a midline skin incision using the EXPEDIUM Spine System (DePuy Synthes, Raynham, MA, USA). After the midline incision and subperiosteal dissection of the erector spine muscles, the affected segment was exposed. Standard laminectomies or extensive decompression was also performed cautiously according to the severity of lumbar degenerative disease. Allograft bone masses were used as the interbody fusion materials at all stabilized segments. Patients in the PLIF group wore a hard lumbar brace for 3 months.

### Clinical and radiographic evaluations

All patients received postoperative clinical and radiographic examinations at 3 months, 1 year, and then every year afterward. The duration of follow-up and surgery, and intraoperative blood loss were retrieved from medical records. Clinical outcomes were evaluated using the Oswestry disability index (ODI) and visual analogue scale (VAS).

Antero-posterior, lateral, and flexion-extension lateral radiographs of the two groups were obtained preoperatively and at each follow-up visit using the digital PACS radiographic imaging review system. Radiographic outcomes included segmental ROM and the disc height of the stabilized segments and upper adjacent segments. Because ASD frequently occurred superior to the operated segment, we only evaluated the radiographic outcomes of the upper adjacent segment [8]. The segmental angulations were measured in the lateral standing lumbar radiography between the inferior surface of the upper vertebra and the superior surface of the lower vertebra. The segmental ROM was calculated as the difference between the segmental angulations in flexion and extension. For multiple operated segments, the segmental ROM was the mean value of all the operated segmental ROMs. We also divided each group into two subgroups (single and multiple level groups) for subgroup analysis of segmental ROM. The disc height was determined on lateral radiographs by calculating the mean of anterior and posterior disc height. The occurrence of radiographic and symptomatic ASD between the two groups was evaluated as described in a previous study [8]. Radiologic ASD was defined as a condition of upper adjacent segment observed by radiology in which the disc height was narrowed by more than 3 mm, the posterior opening observed on flexion lateral radiographs was >5 degrees compared with the preoperative condition, and the progression of slippage was >5% compared with the preoperative flexion and extension lateral radiographs. Symptomatic ASD was defined as the presence of severe radicular pain and cauda equina symptoms from the upper adjacent segment after a period of postoperative relief. The presence of a “double halo” sign on plain radiographs was defined as screw loosening, as described previously [9]. Each radiograph was measured and analyzed independently by two experienced spine surgeons to minimize human error. A senior surgeon resolved disagreements in measurements.

### Statistical analysis

All data were analyzed using SPSS 17.0 statistical software (IBM-SPSS, Inc., Chicago, IL, USA). Continuous variables and categorical data are presented as the mean ± standard deviation (SD) and number, respectively. Preoperative data were evaluated using the Mann-Whitney U test in case of continuous variables and the chi-square/Fisher’s exact test in case of categorical data to ensure that both groups of patients were comparable before surgery. The results of improvement differences from baseline (pre-op) to each follow-up within each group were assessed using the Wilcoxon matched-pairs signed-ranks test. A probability (*P*) value < 0.05 was considered statistically significant.

## Results

Mean patient age, gender, and follow-up time were similar between groups. Patient demographics and baseline characteristics are shown in Table 1. The mean follow-up time in the Dynesys and PILF groups was 53.6 ± 5.3 and 55.2 ± 6.8 months, respectively. The minimum follow-up time was 48 months for each group. Pre-operatively, there were no significant differences between groups in terms of clinical outcomes (VAS and ODI) or radiographic measurements (ROM and disc height). There were no significant differences in the levels of the operated segments or composition of disease between the two groups (Table 1).

**Table 1:** Patient demographic and baseline data.

	Dynesys group (n = 46)	PLIF group (n = 50)	*P*
Age (years)	48.1±12.3	52.3±12.9	0.11*
Gender (male/female)	(31/15)	(37/13)	0.48†
Follow-up time (months)	53.6±5.3	55.2±6.8	0.19*
Operating levels			
Single level	32	35	0.92†
L3/4	2	3	
L4/5	17	19	
L5/S1	13	13	
Multiple levels	14	15	0.81†
L3-L5	3	2	
L4-S1	9	10	
L3-S1	2	3	
Diseases			
Spinal stenosis	20	23	0.80†
Lumbar disc herniation	26	27	
ROM (°)			
stabilized segment	7.1±2.2	7.3±2.3	0.66*
the upper adjacent segment	8.3±2.9	8.5±2.1	0.70*
Disc height (mm)			
stabilized segment	12.0±2.1	12.5±2.3	0.26*
the upper adjacent segment	12.3±1.6	12.9±1.9	0.09*
Surgical duration (min)	103.4±17.9	129.4±26.2	< 0.01*
Intraoperative blood loss (ml)	229.3±56.5	363.4±89.1	< 0.01*

Values are presented as the mean ± standard deviation.

PLIF, posterior lumbar interbody fusion; ODI, Oswestry disability index; VAS, visual analogue scale; ROM, range of motion.

*P* values are based on the Mann-Whitney U test * or chi-square/Fisher’s exact test†.

### Clinical outcomes

The ODI and VAS scores of both groups were significantly improved at the final follow-up as compared to baseline values (*P* < 0.05, Table 2), but the difference at the final follow-up between the two groups was not significant (*P* > 0.05, Table 2). The surgical duration and intraoperative blood loss were both significantly less in the Dynesys group, as compared to those of the PLIF group (103.4 ± 17.9 vs. 129.4 ± 26.2 min; 229.4 ± 56.5 vs. 363.4 ± 89.1 ml, respectively, *P* < 0.05, Table 1).

**Table 2:** Clinical outcomes of the two groups.

	Preoperative	Postoperative	*P*
ODI (%)			
Dynesys group	53.1±13.3*	14.9±5.3*	<0.001†
PLIF group	56.3±16.9*	16.3±6.8*	<0.001†
VAS			
Dynesys group	6.5±2.1*	1.5±0.5*	<0.001†
PLIF group	7.1±2.1*	1.7±0.7*	<0.001†

Values are presented as the mean ± standard deviation.

PLIF, posterior lumbar interbody fusion; ODI, Oswestry disability index; VAS, visual analogue scale.

*No significant difference preoperatively and at the final follow-up between the Dynesys and PLIF groups using the Mann-Whitney U test, *P* > 0.05.

^†^Significant difference between pre- and postoperative condition in each group using the Wilcoxon matched-pairs signed-ranks test, *P* < 0.05.

### Radiographic outcomes

All radiographic outcomes are presented in Table 3. At the final follow-up, the disc height of stabilized segments was slightly increased in the Dynesys group (*P* > 0.05) and significantly increased in the PLIF group (*P* < 0.05), with a significant difference between the two groups (*P* < 0.05). There was no significant decrease in the disc height of the upper segment in either group (*P* > 0.05).

**Table 3:** Radiographic outcomes of the two groups.

	Preoperative	Postoperative	*P*
Disc height of stabilized segment (mm)			
Dynesys group	12.0±2.1	12.6±1.9*	0.15
PLIF group	12.5±2.3	14.1±1.7*	0.01†
Disc height of the upper adjacent segment (mm)			
Dynesys group	12.3±1.6	11.8±1.7	0.14
PLIF group	12.9±1.9	12.3±1.8	0.10
ROM of stabilized segment (°)			
Dynesys group	7.1±2.2	4.9±2.2*	<0.001†
Single level	7.0±2.3	4.8±2.3*	<0.01†
Multiple levels	7.4±2.1	5.1±2.1*	<0.01†
PLIF group	7.3±2.3	0*	<0.001†
Single level	7.1±2.4	0*	<0.01†
Multiple levels	7.5±2.1	0*	<0.01†
ROM of the upper adjacent segment (°)			
Dynesys group	8.3±2.9	10.1±2.7*	0.002†
PLIF group	8.5±2.1	13.1±3.1*	<0.001†

Values are presented as the mean±standard deviation.

PLIF, posterior lumbar interbody fusion; ROM, range of motion.

*Significant difference between the Dynesys and PLIF groups using the Mann-Whitney U test, *P* < 0.05.

^†^Significant difference between pre- and postoperative condition in each group using the Wilcoxon matched-pairs signed-ranks test., *P* < 0.05.

The ROM of stabilized segments in the Dynesys group decreased from 7.1 ± 2.2° to 4.9 ± 2.2° (*P* < 0.05), while that of in the PLIF group decreased from 7.3 ± 2.3° to 0° (*P* < 0.05) at the final follow-ups. The ROM of the upper segment increased significantly in both groups at the final follow-up (*P* < 0.05), but was higher in the PLIF group (13.1 ± 3.1° vs. 10.1 ± 2.7°, *P* < 0.05). Subgroup analysis showed that the number of operated levels (single and multiple levels) did not affect the results (Table 3). The fusion rate was 92.0% (46/50) in the PLIF group at the final follow up, however these instruments were stable and the ROMs of the stabilized segments were still 0°.

**Fig 1:**
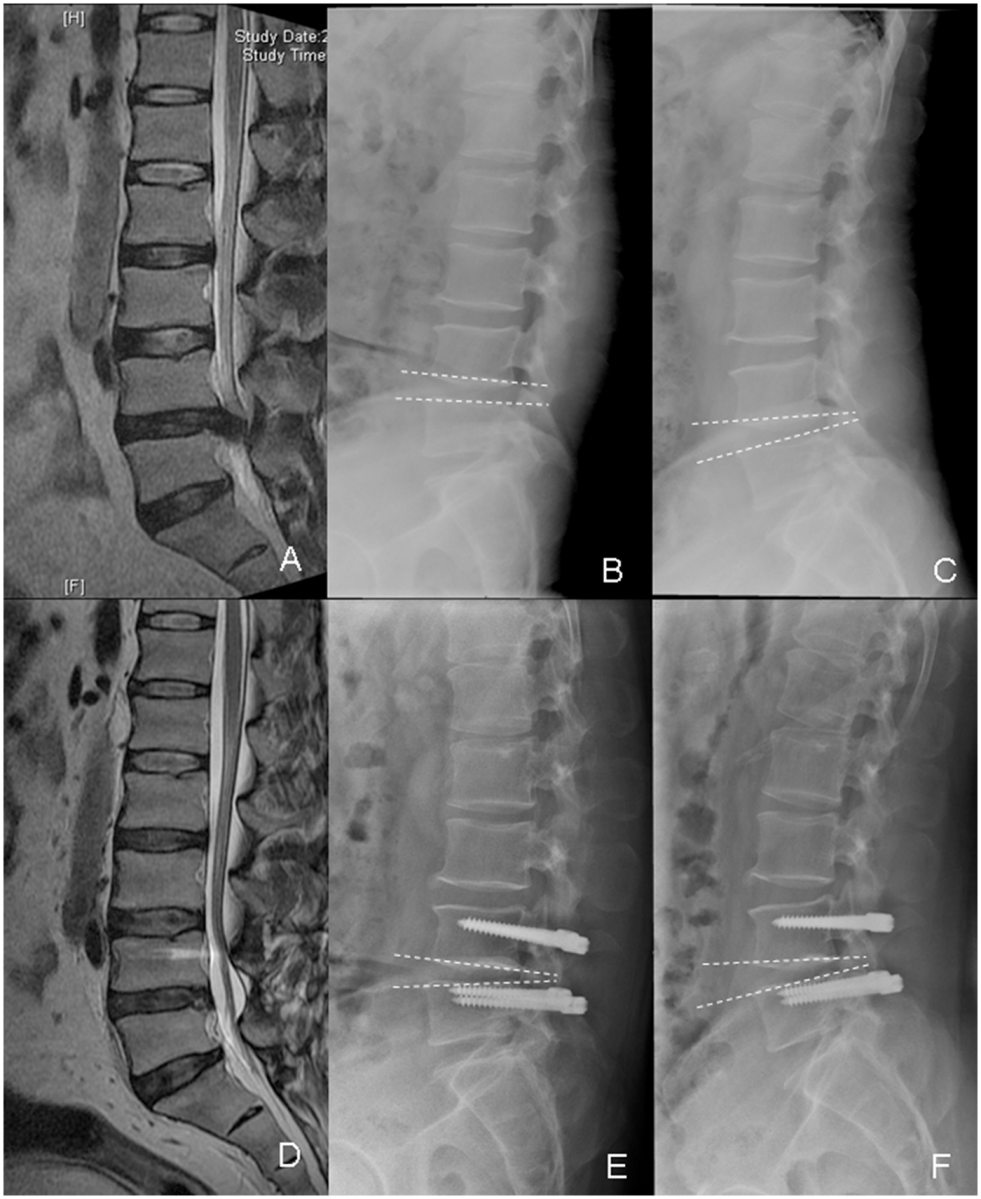
The radiological data of the patients with lumbar disc herniation in the Dynesys group. A 48-year-old male patient underwent Dynesys stabilization due to lumbar disc herniation in L4/5. A: The preoperative lumbar MRI. B-C: The preoperative flexion and extension X-rays, the ROM of L4/5 was 6°; D: The lumbar MRI at 36 months after the operation; E-F: The flexion and extension X-rays 48 months after the operation, the ROM of L4/5 was 4°.ROM: range of motion.

**Fig 2:**
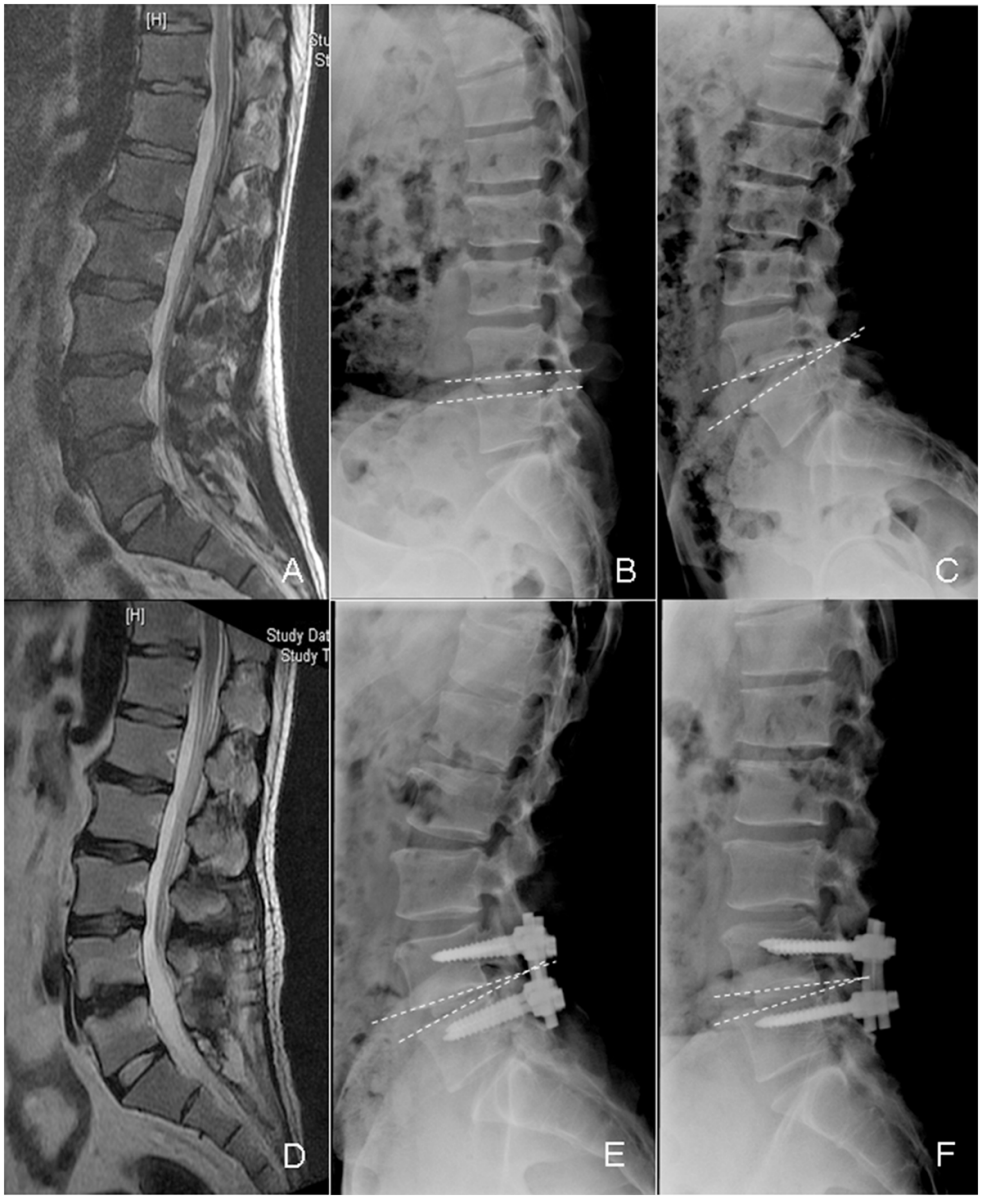
The radiological data of the patients with lumbar disc herniation in the PLIF group. A 44-year-old male patient underwent PLIF due to lumbar disc herniation in L4/5. A: The preoperative lumbar MRI. B-C: The preoperative flexion and extension X-rays, the ROM of L4/5 was 12°; D: The lumbar MRI at 50 months after the operation; E-F: The flexion and extension X-rays 50 months after the operation, the ROM of L4/5 was 0°.ROM: range of motion; PLIF: posterior lumbar interbody fusion.

There were significantly more radiographic ASDs in the PLIF group than in the Dynesys group (15 vs. 6, respectively; χ^2^ = 4.13, *P* < 0.05). Only one symptomatic ASD occurred in the PLIF group, and the patient underwent a second operation. The radiographs and MRI of typical patients in both groups are shown in Figs 1 and 2, respectively.

### Complications

There were no significant differences in the incidence of asymptomatic screw loosening between the Dynesys and PLIF groups (6 vs. 8, respectively; χ^2^ = 0.17, *P* = 0.68). There was no instance of breakage in either group at the final follow-up. One iatrogenic dural tear occurred intraoperatively in the Dynesys group and two in the PLIF group, which were all managed without further complications. There was one case of superficial wound infection in the Dynesys group and three in the PLIF group, which were treated by conservative therapy. There were no other complications or reoperation in either group.

## Discussion

The result of this study indicated that after a minimum follow-up of 4 years, both Dynesys and PLIF improved clinical outcomes for lumbar degenerative disease. Compared to PLIF, Dynesys stabilization partially preserved the ROM of the stabilized segments and limited the increase in the ROM at the upper adjacent segment.

Adjacent segment disease after spinal fusion has drawn considerable attention over the past two decades. Spinal fusion results in increased stress in the adjacent segments and subsequent hypermobility, which may leads to adjacent segment disease[10, 11]. This problem can be resolved by the development of dynamic stabilization techniques. Stoll et al. [12] suggested that the preservation of ROM at the stabilized segment can prevent degeneration at the adjacent segments by decreasing stress and preventing hypermobility. The Dynesys system is designed with the intention to neutralize abnormal forces and restore painless function to the spinal segments, while protecting the adjacent segments. Schmoelz et al. [13] suggested that the Dynesys system provided substantial stability in case of degenerative spinal pathologies and can therefore be considered as an alternative method to fusion surgery in these indications, while preserving segment motion.

Many studies have reported positive outcomes for patients treated with the Dynesys system [4, 5, 14]. Welch et al. [15] optimistically reported preliminary results from the Food and Drug Administration investigational device exemption clinical trial of the Dynesys system. They noted that the Dynesys system might be preferable to lumbar fusion for degenerative lumbar disease because it decreased back and leg pain. Yu et al. [6] compared 35 patients who received Dynesys at three segments with 25 patients with the same indications who underwent three-level PLIF and found greater improvement in ODI and VAS in the Dynesys group than the PLIF group at 3-year follow-ups.

However, some studies have indicated that the clinical results with the Dynesys system are no better than those after PLIF. Legaye et al. [16] found that the Dynesys system might result in the loss of lordosis, which is a cause of excessive mechanical stress on the lumbar structures, and could lead to long-term degradation. A randomized controlled study performed by Yu et al. [17] noted that the improvements in ODI and VAS were similar between the Dynesys and PLIF groups for the treatment of lumbar spinal stenosis at L4/5 at 3-year follow-ups. Haddad et al. [18] retrospectively compared clinical outcomes between the Dynesys system and PLIF, and found that after 4 years, VAS for back and leg pain and ODI improved significantly in both groups, with all scores better in the PLIF group.

In the present study, the ODI and VAS were significantly improved in both groups at the final follow-ups; however, the differences between the two groups were not significant. We concluded that the Dynesys system was an acceptable alternative to PLIF for the treatment of lumbar degenerative disease. The surgical duration and intraoperative blood loss were both significantly less in the Dynesys group, as compared to those in the PLIF group. The reason for the shorter surgical duration in the Dynesys group was that there was no need for endplate and autogenous bone preparation or bone grafting. The lower blood loss in the Dynesys group was mainly because the screws were placed via the Wiltse approach and there was less bone and soft tissue dissection [19].

Although the Dynesys system has been available for more than 10 years, it remains controversial whether this dynamic stabilization system can prevent the occurrence of ASD [20–22]. Two studies by Yu et al. [6, 17] noted that as compared to PLIF, Dynesys stabilization resulted in significantly higher preservation of ROM at the stabilized segments and significantly less hypermobility at the adjacent segments. Beastall et al. [23] analyzed 24 patients treated with the Dynesys system and at 9 months postoperatively they found limited movement at the stabilized segment and no significant increase in mobility at the adjacent segments. However, Schaeren et al. [14] found that after a minimum 4-year follow-up, there was no measurable motion at the operative segment with Dynesys stabilization, while new signs of degeneration were present in adjacent segments in 47% of the patients. Cakir et al. [10] reported that after a minimum follow-up of 24 months, neither monosegmental PLIF nor Dynesys system altered the ROM at the cranial or caudal adjacent levels. They also noted that only the segmental ROM at the index level decreased significantly in the PLIF group, while there was no significant difference in the Dynesys group.

The results of the current study showed that Dynesys stabilization partially preserved the ROM of the stabilized segments, while that in the PLIF group decreased to zero degrees. Also, subgroup analysis of segmental ROM according to the operated levels (single level and multiple levels) showed that these results were similar to the main results. The ROM of the upper segments increased significantly in both groups at the final follow-up, although to a higher degree in the PLIF group. The reason for the lower instance of hypermobility in the upper adjacent segments in the Dynesys group was likely because the Dynesys system could share the load in the operative segment and did not convey an excessive load to the adjacent segments. The occurrence of radiographic ASDs was significantly lower in the Dynesys group as compared to that in the PLIF group, which might be mainly attributable to the dynamic stabilization of the operated segments and the avoidance of increased stress at the adjacent segment.

The complications were comparable between the two groups. In our study, asymptomatic screw loosening was confirmed by the presence of a “double halo sign” on plain radiographs. Ko et al. [24] reported screw loosening in 19.7% of patients and 4.6% of screws after Dynesys stabilization for 1- and 2-level lumbar spondylosis at a mean follow-up of 16.6 months. In the present study, the incidence of asymptomatic screw loosening was 13.0% (6/46) in the Dynesys group and 16.0% (8/50) in the PLIF group, indicating no significant difference. Nonetheless, screw loosening has no adverse effect on clinical improvement. The ODI and VAS scores of the patients with screw loosening were similar to those of the patients without (see S2 Table). Furthermore, the fact that Dynesys screws were coated with hydroxyapatite was an attempt to prevent screw loosening. The incidence of other complications, including dural tear and superficial wound infection, was similar between groups.

## Conclusion

Both the Dynesys system and PLIF can improve clinical outcomes of lumbar degenerative disease. Compared to PLIF, Dynesys stabilization partially preserves the ROM of the stabilized segments, limits hypermobility in the upper adjacent segment, and may prevent the occurrence of ASD.

## Supporting Information

S1 Table Original data of this study. (XLS)

S2 Table Final clinical outcomes of patients with screw loosening. (XLS)
